# Six steps for building a technological knowledge base for future taxonomic work

**DOI:** 10.1093/nsr/nwac284

**Published:** 2022-12-15

**Authors:** Michael C Orr, Anderson Feijó, Douglas Chesters, Alfried P Vogler, Silas Bossert, Rafael R Ferrari, Mark John Costello, Alice C Hughes, Lars Krogmann, John S Ascher, Xin Zhou, De-Zhu Li, Ming Bai, Jun Chen, Deyan Ge, Arong Luo, Gexia Qiao, Paul H Williams, Ai-bing Zhang, Keping Ma, Feng Zhang, Chao-Dong Zhu

**Affiliations:** Key Laboratory of Zoological Systematics and Evolution, Institute of Zoology, Chinese Academy of Sciences, China; Entomologie, Staatliches Museum für Naturkunde Stuttgart, Germany; Key Laboratory of Zoological Systematics and Evolution, Institute of Zoology, Chinese Academy of Sciences, China; Key Laboratory of Zoological Systematics and Evolution, Institute of Zoology, Chinese Academy of Sciences, China; Department of Life Sciences, Silwood Park Campus, Imperial College London, UK; Natural History Museum, UK; Department of Entomology, Washington State University, USA; Department of Entomology, National Museum of Natural History, Smithsonian Institution, USA; Key Laboratory of Zoological Systematics and Evolution, Institute of Zoology, Chinese Academy of Sciences, China; Faculty of Biosciences and Aquaculture, Nord University, Norway; School of Biological Sciences, University of Hong Kong, China; Entomologie, Staatliches Museum für Naturkunde Stuttgart, Germany; Department of Biological Sciences, National University of Singapore, Singapore; Department of Entomology, China Agricultural University, China; Germplasm Bank of Wild Species, Kunming Institute of Botany, Chinese Academy of Sciences, China; Key Laboratory of Zoological Systematics and Evolution, Institute of Zoology, Chinese Academy of Sciences, China; Key Laboratory of Zoological Systematics and Evolution, Institute of Zoology, Chinese Academy of Sciences, China; Key Laboratory of Zoological Systematics and Evolution, Institute of Zoology, Chinese Academy of Sciences, China; Key Laboratory of Zoological Systematics and Evolution, Institute of Zoology, Chinese Academy of Sciences, China; Key Laboratory of Zoological Systematics and Evolution, Institute of Zoology, Chinese Academy of Sciences, China; Natural History Museum, UK; College of Life Sciences, Capital Normal University, China; Institute of Botany, Chinese Academy of Sciences, China; Department of Entomology, College of Plant Protection, Nanjing Agricultural University, China; Key Laboratory of Zoological Systematics and Evolution, Institute of Zoology, Chinese Academy of Sciences, China; State Key Laboratory of Integrated Pest Management, Institute of Zoology, Chinese Academy of Sciences, China; College of Life Sciences, University of Chinese Academy of Sciences, China

Species are the principal category we use to study, understand, and conserve the natural world, so how we define species has a cascading impact across fields, well beyond biology [1]. Despite such importance, debate on species concepts continues to this day ([2] 2020), with consequences for what organisms we recognize, study and protect. Given the wealth of life forms on our planet, achieving a universally-accepted concept seems impossible. If taxonomists wait for a universally-accepted concept, delaying their work on describing and delimiting species, we risk losing species to extinction before they are named [3].

Thankfully, taxonomists have been describing species for centuries [4], so a universally-accepted definition for species is clearly not a prerequisite for taxonomic research. This is reflected in the unified species concept of De Queiroz [5], which separates the conceptual basis of what the word species mean from the operational methods for delimiting them. Thus, taxonomists can act largely free of the daunting philosophical basis of the word species. But standardized practices in delimitation are still necessary to better our operational bases and improve transparency, so we can clearly communicate species and enable their effective identification. Too often lack of funding, unpublished data, etc. prevent the use of multiple data types, even though they perform more accurately for identification when used together (potentially automated, [6]). To further standardize efforts, across taxonomy and for specific organismal groups, we need comparable data and methodology.

To truly maximize the value of diverse data sources, we must make every effort to link specimens to the formal species names that are defined by taxonomists in order to unify biological research [7,8], rather than strictly operational units. Otherwise, we risk re-inventing the wheel, building a parallel system of such units largely disconnected from both centuries of prior research and conservation efforts made by organizations such as the IUCN and CITES, which often legally function using species names [1].

Authoritative species catalogs, image databases (especially of type specimens), and DNA reference libraries are keys to this puzzle [4,6,7]. Therefore, specimens should, wherever possible, be leveraged for (1) molecular data, (2) morphological data (images, etc.) and traits, (3) geographic information, and (4) identifier history. Without these, we cannot determine what are known versus unknown species, nor can we effectively build and share our knowledge of described species. Further, to reliably delimit species and assess their conservation status we must adequately incorporate variation, as a single collection per species remains insufficient for many purposes. For more effective conservation of some of the most species-rich groups, active synergy across fields is needed [3].

**Figure 1.**
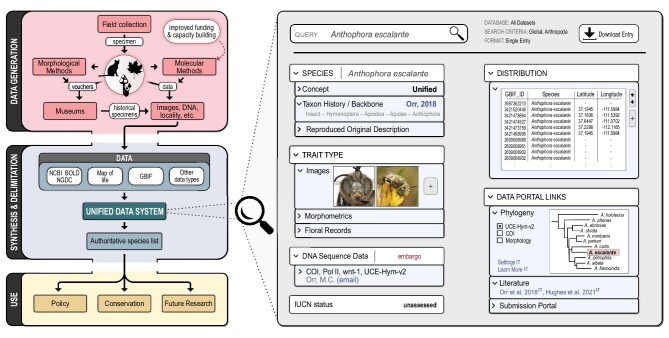
Workflow for synergizing overarching specimen data. Where data come from, is stored, and its subsequent usage. The full workflow is given on the left, with an exemplary species-level query to the unified data system on the right. Note that many arrows may become bidirectional with feedback loops. Face image taken from Ref. [9]. Displayed distribution data are from GBIF (https://doi.org/10.15468/dl.b363m8).

To this end, we suggest a set of best practices and solutions:

1. Vouchers and standards. All studies involving specimen collection, with cross-referenced tissues for DNA, must minimally deposit vouchers in collections [8]. Resulting specimens, and historically valuable specimens like types, should be imaged and made publicly available. Likewise, taxonomic revisions should incorporate morphological, molecular, and other available evidence, including verifying or flagging public data as problematic in order to eliminate misidentification. Within groups, standards should be agreed upon for the specific tissues to preserve, uniform measurements expected and imaging parameters.
2. Data standards and cross-operability. Deposition of new specimen data should mandate an identifier field to indicate the taxonomic authority as a measure of reliability (including how they were identified, which is especially valuable for replicability). Making identifications versionable and historically traceable will enable multiple experts to endorse identifications or flag them as problematic. Similarly, location data at appropriate scales should be mandated. When a new sequence is uploaded to a molecular repository, it should automatically be generated as a specially flagged record on GBIF (traced to extant records and versionable cross-platforms via unique identifiers; initial links are already being made, see https://www.gbif.org/dna).
3. A singular data system. We envision a unified platform with a centralized system integrating extant databases and including other diverse data types such as pertinent literature, conservation and protection status, species concept applied, etc. (Fig. 1; [3]). Some repositories have attempted this but remain incipient at best. Central to this is a unified species list such as Catalogue of Life [4,7], but disagreements based on differing species concepts should be incorporated (as begun in the World Register of Marine Species), all directed hand-in-hand with taxonomists.
4. Species concept justification. Descriptions of new species must be as comprehensive as possible, integrating all available data. Since these data may disagree in species delineations (for example, DNA and morphology), we must explicitly state the justifications of new species designations and lines of evidence employed in revisions and elsewhere, including how they have been integrated. This will greatly improve both transparency and reproducibility in taxonomy.
5. Increased automation throughout. Generalized workflows can be created by incorporating public sequence data and species delimitation methods [6], and these can be hosted online, like BOLD’s BIN system for short DNA barcodes, but similar public data systems remain unrealized for many other data types. Potential new species may be identified automatically from these data, thus better enabling integrative taxonomic approaches [10], based on continually-refined reference libraries leveraging verified historical specimens’ images and DNA sequences.
6. Representative sequences for poorly-known groups. As a preliminary step before full species description, ‘type’ sequences could be designated (similar to NCBI RefSeqs). As single markers can be problematic (pseudogenes, incomplete lineage sorting), multiple markers are preferred. These preliminary ‘species’ could be used for conservation assessment and restoration until official species descriptions are available.

Many of these steps presuppose funding, but just as important are enhanced supports and more relevant career assessments that better value data generation and species description [3]. In support of recent pushes for open data, funding agencies should stipulate that relevant molecular and morphological data are made public on firm timelines with standard approaches (not ‘available on reasonable request’). This has recently been suggested by the National Institute of Health and should be adopted more broadly [11]. With a sliding scale of expectation, depending on career stage and country, such policies could be made more equitable for those disproportionately impacted by regulations [3]. In making research equitable across fields and countries, we can truly optimize synergies and outcomes for biodiversity and those who study it.
